# Online revenue maximization for server pricing

**DOI:** 10.1007/s10458-022-09544-y

**Published:** 2022-01-22

**Authors:** Shant Boodaghians, Federico Fusco, Stefano Leonardi, Yishay Mansour, Ruta Mehta

**Affiliations:** 1 University of Illinois at Urbana-Champaign, Urbana, IL 61801 USA; 2 Department of Computer, Control and Management Engineering, Sapienza University, 00185 Rome, Italy; 3 Tel Aviv University, P.O. Box 39040, 6997801 Tel Aviv, Israel

**Keywords:** Server pricing, Markov Decision Process, Pricing

## Abstract

Efficient and truthful mechanisms to price resources on servers/machines have been the subject of much work in recent years due to the importance of the cloud market. This paper considers revenue maximization in the online stochastic setting with non-preemptive jobs and a unit capacity server. One agent/job arrives at every time step, with parameters drawn from the underlying distribution. We design a posted-price mechanism which can be efficiently computed and is revenue-optimal in expectation and in retrospect, up to additive error. The prices are posted prior to learning the agent’s type, and the computed pricing scheme is deterministic, depending only on the length of the allotted time interval and on the earliest time the server is available. We also prove that the proposed pricing strategy is robust to imprecise knowledge of the job distribution and that a distribution learned from polynomially many samples is sufficient to obtain a near-optimal truthful pricing strategy.

## Introduction

Designing mechanisms for a desired outcome with strategic and selfish agents is an extensively studied problem in economics, with classic work by Myerson [30], and Vickrey–Clarke–Groves [37], for truthful mechanisms. The advent of online interaction and e-commerce has added an efficiency constraint on the mechanisms, going so far as to prioritize computational efficiency over classical objectives: e.g., choosing simple approximate mechanisms when optimal mechanisms are computationally difficult, or impossible. Beginning with Nisan and Ronen [31], the theoretical computer science community has contributed greatly to the field, in both fundamental problems and specific applications. These include designing truthful mechanisms for the maximization of welfare and revenue, and has also focused on learning distributions of agent types, menu complexity, and dynamic mechanisms (*e.g.*, [10, 13]).

We consider this question in the setting of selling computational resources on remote servers or machines (*cf.* [2, 36]). This is arguably one of the fastest growing markets on the Internet. The goods (resources) are assigned non-preemptively and thus have strong complementarities. Furthermore, since the supply (server capacity) is limited, any mechanism trades immediate revenue for future supply. Finally, mechanisms must be incentive-compatible, as non-truthful, strategic, behaviour from the agents can skew the performance of a mechanism from its theoretical guarantees. This leads us to the following question:

*Can we design an efficient, truthful, and revenue-maximizing mechanism to sell time-slots non-preemptively on a single server?*

We design a posted-price mechanism which maximizes the expected revenue up to additive error, for agents/buyers arriving online, with parameters of value, length and maximum delay drawn from the underlying distribution. Three key aspects distinguish our problem from standard online scheduling:

1. In our setting, as time progresses, the server clears up, allowing longer jobs to be scheduled in the future if no smaller jobs are scheduled until then.
2. Scheduling the jobs is not exclusively to the discretion of the mechanism designer, but must also be desired by the job itself, while also producing sufficient revenue.
3. As the mechanism designer, we do not have access to job parameters in an incentive-compatible way before deciding on a posted price menu.

These three features lie at the core of the difficulty of our problem. Our focus will be on devising online mechanisms in the Bayesian setting.

In our online model, time on the server is discrete. At every time step, an agent arrives at the server with a value *V*, length requirement *L*, and maximum delay *D*. These parameters are drawn from a common distribution, *i.i.d.* across jobs. The job wishes to be scheduled for at least *L* consecutive time slots, no more than *D* time units after its arrival, and wishes to pay no more than *V*. Jobs are assumed to be rational agents having quasi-linear utility in money, therefore they prefer the least-price interval within their constraints. The mechanism designer never learns the parameters of the job. Instead, she posts a price menu of (length, price) pairs, and the minimum available delay *s*. The job accepts to be scheduled as long as $D\ge s$, and there is some (length, price) pair in the menu of length at least *L* and price at most *V*. We note that the pricing scheme can be dynamic, changing through time. If, at time epoch *t*, an agent chooses option $(\ell ,\pi _\ell )$, then she pays $\pi _\ell$ and her job will be allocated to the interval $[t+s,t+s+\ell ]$. She will choose the option which minimizes $\pi _\ell$. Throughout this paper, we assume that the random variables *L*, *V*, *D* are discrete and have finite support, unless specified differently.

### Summary of our results

1. We model the problem of finding a revenue maximizing pricing strategy as a Markov Decision Process (MDP). Given a price menu (length, price) and a state (minimum available delay) *s* at time *t*, the probability of transition to any other state at time $t+1$ is obtained from the distribution of the job’s parameters. The revenue maximizing pricing strategy can be efficiently computed via backwards induction. We also present an approximation scheme in the case where *V* is a continuous random variable.
2. We prove that the optimal pricing strategy is monotone in length under a distributional assumption, which we show is satisfied when the jobs’ valuation follows a log-concave distribution, parametrized by length. Recall that log-concave distributions are exactly those which have a monotone hazard rate. This implies the existence of an optimal pricing mechanism which ensures truthfulness in the finite horizon setting when the distributions are known. This is extended to the infinite discounted horizon setting, incurring a small additive error. We also demonstrate good concentration bounds of the revenue obtained by the optimal truthful posted price strategy.
3. We finally investigate the robustness of the pricing strategy. We first show that a near optimal solution is still obtained when the distribution is known with a certain degree of uncertainty. We complement this result by analyzing the performances of the proposed pricing strategy when the distribution is only known from samples collected through the observations of the agents’ decisions. We provide a truthful posted price $\varepsilon$-approximate mechanism if the number of samples is polynomial in $1/\varepsilon$ and the size of the support of the distribution.

### Related work

A good starting point for a review of the revenue maximization in the cloud market literature is given by the paper of Kash and Key [21]. It offers a thorough discussion of the challenges and different dimensions of complexity of the problem, with pointers to the related literature. For comparison, our model falls within their unidimensional offering framework, since each job is only interested in getting allocated in its entirety paying a price as low as possible.

Probably the work closest to ours is Kash et al. [22]. There the authors studied the steady state of a stochastic process in which i.i.d. jobs arrive at discrete time steps, each job is characterized by a length and a value-per-unit that are drawn independently from two known distributions. The main result of the paper is the design of a price-per-unit scheme that approximates within a multiplicative factor of 2 the revenue (or the welfare) extracted by the best price menu. This work has three main differences from ours: first, their focus is on the simple vs optimal side of the problem (i.e., fixed price-per-unit vs optimal price menus), second we consider the additional constraint given by the deadlines and finally in our model the jobs are drawn from a possibly correlated distribution (a feature which increases the complexity of the pricing problem). Kash et al. [22] also showed how to generalize their results for multiple servers, this is also an interesting direction for further work in our model.

Another line of work studies the game-theoretical problem of adopting pricing strategies when multiple server providers (or different services within the same provider) compete for the jobs. Dierks and Seuken [11] investigated the interplay between two types of services offered by server providers; i.e., the main market where computing time is sold at a fixed price and the spot market where the resources left unused by the main market are priced dynamically. The same authors studied in [12] the competition between fixed price-per-unit strategies with respect to type-dependent ones, characterizing the Bayesian Nash Equilibria of a market where different server providers use the two strategies. Kash et al. [23] considered the problem faced by a server provider that is willing to periodically adopt new technologies and has to devise a pricing strategy that takes into consideration the switching cost of its clients from the old contract to the new ones.

We also mention two more applied papers: Kilcioglu et al. [24] addressed the problem of computing a price menu for revenue maximization with different machines and offered extensive numerical experiments. Babaioff et al. [2] proposed a system architecture for scheduling and pricing in cloud computing.

Revenue maximization is not the only objective considered in a mechanism design perspective of the cloud pricing problem. Azar et al. [1] provided a mechanism for preemptive scheduling with deadlines maximizing the total value of completed jobs (i.e., welfare). Chawla et al. [8] studied “time-of-use” pricing mechanisms, to match demand to supply with deadlines and online arrivals. They assume large-capacity servers, while seeking to maximize welfare in a setting in which the jobs arriving over time are not i.i.d.

Another possible objective for the design of incentive-compatible scheduling mechanisms is the total value of completed jobs, which have release times and deadlines. Porter [32] solved this problem in an online setting, while Carroll et al. [6] did the same in the offline setting for parallel machines, and Ströle et al. [35] in the online competitive setting with uncertain supply. Jain et al. [20] focused on social welfare maximization for non-preemptive scheduling on multiple servers and obtained a constant competitive ratio as the number of servers increases.

Note that revenue maximization is arguably a harder objective than welfare or total allocated time. As an example, consider a simplified instance where the jobs have all unitary length and the mechanism has to *learn* the underlying distribution by posting prices (as we do in Sect. 4.2); the welfare maximizing strategy ignores the learning problem and *always* accepts the arriving job, e.g., setting the price to 0, while a revenue maximizing mechanism would need to identify Myerson’s reserve value. Although our focus is on a different objective, we nevertheless share many modelling assumptions with the welfare maximization papers: we also consider posted prices, stochastic jobs and assume each job to be characterized by length, value, arrival time and internal deadline.

Posted price mechanisms (PPM) have been introduced by Sandholm et al. [34] and have gained attention due to their simplicity, robustness to collusion, and their ease of implementation in practice. One of the first theoretical results concerning PPM’s is an asymptotic comparison to classical single-parameter mechanisms by Blumrosen et al. [5]. They were later studied by Chawla et al. [9] for the objective of revenue maximization, and further strengthened by Kleinberg et al. [25] and Dütting et al. [15]. Feldman et al. [16] showed that sequential PPM’s can $\frac{1}{2}$-approximate social welfare for XOS valuation functions, if the price for an item is equal to the expected contribution of the item to the social welfare. Recently, Dütting et al. showed how simple PPMs outperform the best known complex mechanisms in double auctions [14].

The intrinsic truthfulness of posted price mechanisms and the specific structure of the feedback received by the mechanism (i.e., the trade happens or not given the posted prices) have been investigated extensively in the online learning community, starting from the seminal work of Kleinberg and Leighton [26]. We refer the interested reader to the survey by den Boer [13] and the more recent paper by Cesa-Bianchi et al. [7] for additional related work. Notice that this type of feedback problems are similar to the ones we encounter in our problem: posting a price menu and observing that a job of length $\ell$ gets scheduled for price *p* let us infer that the value of the sampled job is larger than *p*, but does not tell us its exact value.

The systematic study of sample complexity of revenue maximizing auctions has been initiated in the seminal work of Morgenstern and Roughgarden [28]. In a follow-up paper from the same authors, simple auctions have also been analyzed [29]. Sample complexity for revenue maximization has recently been studied by Cole et al. [10] showing that polynomially many samples suffice to obtain near optimal Bayesian auction mechanisms. A generalization to multidimensional auctions has recently appeared in [18]; we also mention [19] that exhibits tight bounds on the sample complexity of many problems related to mechanism design.

### Structure of the paper

In Sect. 2 we describe the problem as a Markov Decision Process. In Sect. 3 we present an efficient algorithm for computing optimal policies for the finite time horizon given full knowledge of the distribution of the jobs’ parameters. This is then extended to other settings in Sects. 3.3 and 3.4.

In Sect. 3.5, we demonstrate that the optimal policy is monotone and in  Sect. 3.6 we describe the concentration bounds on the revenue of a pricing policy. Sections 4.1 and 4.2 give the learning algorithm and error bounds for computing the pricing policies with only (partial) sample access to the job distribution.

Finally, Sects. 4.3 and 5 are devoted to describing and summarizing the achieved results and future directions of research.

## Model

*Notation* In what follows, the variables *t*, $\ell$ or *L*, *v* or *V*, and *d* or *D* are reserved for describing the parameters of a job that wishes to be scheduled. Respectively, they represent the arrival time *t*, required length $\ell$, value *v*, and maximum allowed delay *d*. The lowercase variables represent fixed values, whereas the uppercase represent random variables. Script-uppercase letters $\mathcal L,\mathcal V,\mathcal D$ represent the supports of the distributions on *L*, *V*, and *D*, respectively; and the bold-uppercase letters $\mathbb L,\mathbb V,\mathbb D$ represent the maximum values in these respective sets. Finally, $\pi$ is reserved for pricing policy, whereas *p* is reserved for probabilities.

*Single-machine, non-preemptive, job scheduling* A sequence of random jobs wish to be scheduled on a server non-preemptively, for a sufficiently low price, within a time constraint. Formally, at every time step *t*, a single job with parameters (*L*, *V*, *D*) is drawn from an underlying distribution *Q* over the space $\mathcal L\times \mathcal V\times \mathcal D$. It wishes to be scheduled for a price $\pi \le V$ in an interval [*a*, *b*] such that $a-t\le D$ and $b-a\ge L$.

*Price menus* Our goal is to design a take-it-or-leave-it, posted-price mechanism which maximizes the expected revenue. At each time period, the mechanism posts a “price menu” that specifies $\mathbb L$ prices (i.e., one for each one of the $\mathbb L$ possible lengths of the next job) and an earliest-available-time $s_t$, indicating that times *t* through $t + s_t - 1$ have already been scheduled ($s_t$ will henceforth be referred to as the *state* of the server). We let $\mathcal S:= \{0,\,\dotsc ,\,\mathbb D+\mathbb L\}$ to be the set of all possible states. The state of the server at a given time *t* is naturally a random variable which depends on the earlier jobs and on the adopted policy $\pi$. As before, we will denote with *s* or $s_t$ the fixed value, and with *S* or $S_t$ the corresponding random variable. The price menu will be given by $\pi$, a function of the current time, the state of the server and the length of the job arriving. So if we are at time *t* and the server is in state $s_t$, then the prices are set according to $\pi _t(s_t,\cdot ): \mathcal L\rightarrow \mathbb R.$ The reported pair $(\pi _t(s_t,\cdot ),s_t)$ is computed by the scheduler’s strategy, which we determine in this paper. Once this is posted, a job (*L*, *V*, *D*) is then sampled *i.i.d.* from the underlying distribution *Q*.

If $V\ge \pi _t(s_t,\ell )$ for some $\ell \ge L$, and $D\ge s_t$, then the job accepts the schedule, and reports the length $\ell \ge L$ which minimizes price. Otherwise, the job reports $\ell =0$ and is not scheduled. To guarantee truthfulness, it suffices to have $\pi _t(s,\cdot )$ monotonically non-decreasing for every state *s*: the agent would not want a longer interval since it costs more, and would not want one of the shorter intervals since they cannot run the job. It should be clear that the mechanism’s strategy is to always report monotone nondecreasing prices, as a decrease in the price menu will only cause more utilization of the server, without accruing more revenue. The main technical challenge in this paper, then, is to show that under some assumptions, the optimal strategy is monotone nondecreasing, and efficiently computable.

*Revenue objective* Revenue can be measured in either a *finite* or an *infinite discounted* horizon. In the finite case, only *T* time periods occur and we seek to maximize the expected sum of revenue over these periods. Recall that the job parameters are drawn independently at random from the underlying distribution, so the scheduler can only base the “price menu” on the state of the system and the current time. Thus, the only realistic strategy is to fix a state-and-time-dependent pricing policy $\pi : [T]\times \mathcal S\times \mathcal L\rightarrow \mathbb R$, “$\pi _t(s,\ell )$”, where $[T]:=\{0,\,1,\,\dotsc ,\,T\}$.

Let ${\mathcal {X}}=\{\mathcal X_1:=(1,L_1,V_1,D_1),\,\mathcal X_2:=(2,L_2,V_2,D_2),\,\mathcal X_3,\,\dotsc \}$ be the random sequence of jobs arriving, sampled *i.i.d.* from the underlying distribution. Let $\pi :[T]\times \mathcal S\times \mathcal L\rightarrow \mathbb R$ be the pricing policy. We denote as $\textsf {Rev}_t({\mathcal {X}},\pi )$ the revenue earned at time *t* with policy $\pi$ and sequence ${\mathcal {X}}$. If $\mathcal X_t$ does not buy, then $\textsf {Rev}_t({\mathcal {X}},\pi )=0$, and otherwise, it is equal to $\pi _t(s_t,L_t)$. We denote as $\textsf {CmlRev}_T$ the total (cumulative) revenue earned over the *T* periods. Thus,

1 $$\begin{aligned} \textsf {CmlRev}_T({\mathcal {X}},\pi ):=\textstyle \sum _{t=0}^T \textsf {Rev}_t({\mathcal {X}},\pi ). \end{aligned}$$

The expected-future-revenue, given a current time and server state, is denoted with:

2 $$\begin{aligned} {U^\pi _{t}(s)}=\mathbb {E}_{\mathcal X_{\ge t}}\left[ \textstyle \sum _{i=t}^T \textsf {Rev}_i(\pi ,\mathcal X) \big | S_t=s \right] , \end{aligned}$$

The subscript of the expectation $\mathcal X_{\ge t}$ denotes that we consider only jobs arriving from time *t* onward. Our objective is to find the pricing policy $\pi$ which maximizes ${U^\pi _{0}(s=0)}$. Call this $\pi ^*$, and denote the expected revenue under $\pi ^*$ as ${U^*_{t}(\cdot )}$.

In the infinite horizon setting, the future revenue is discounted at an exponentially decaying rate. Formally, revenue at time *t* is worth a $\gamma ^t$ fraction of revenue at time 0, for some fixed $\gamma <1$. More precisely, we seek to maximize the $\gamma$-discounted future revenue,

$$\begin{aligned} \textsf {CmlRev}_{\infty }({\mathcal {X}},\pi ):=\sum _{t=0}^{\infty } \gamma ^t\textsf {Rev}_t({\mathcal {X}},\pi ) \end{aligned}$$

over the choice of $\pi :\mathbb N\times \mathcal S\times \mathcal L\rightarrow \mathbb R$.

## Bayes-optimal strategies for sever pricing

In this section, we seek to compute an optimal monotone pricing policy $\pi :[T] \times \mathcal S\times \mathcal L\rightarrow \mathbb R$ which maximizes revenue in expectation over *T* jobs sampled *i.i.d.* from an underlying known distribution *Q*. We also extend this result in two directions: in the infinite-horizon discounted case in Sect. 3.3 and for jobs whose value is distributed continuously in Sect. 3.4.

We first model the problem of maximizing the revenue in online server pricing as a Markov Decision Process that admits an efficiently computable, optimal pricing strategy. The main contribution of this section is to show that, for a natural assumption on the distribution *Q*, the optimal policy is monotone. We recall that this allows us to derive truthful Bayes-optimal mechanisms.

### Markov Decision Processes

The theory of *Markov Decision Processes* is well suited to model our problem. A Markov Decision Process is, in its essence, a Markov Chain whose transition probabilities depend on the *action* chosen at each state, and where to each transition is assigned a reward. A *policy* is then a function $\pi$ mapping states to actions. In our setting, the states of the system are the ones outlined in Sect. 2 (i.e., the possible delays before the earliest available time on the server), and the actions are the “price menus.” At every state *s*, a job arrives, and according to its random features and the prices offered, is scheduled. The next state is either $\max \{s-1,0\}$, if the job does not choose to be scheduled (since we have moved forward in time), or $s+\ell -1$, if a job of length $\ell$ is scheduled, since we have occupied $\ell$ more units. The transition probabilities depend on the distribution of job lengths, and the probability that a job accepts to be scheduled given the pricing policy (action). Formally,

3 $$\begin{aligned} \mathbb {P}[s_{t+1}=s_t+\ell -1] = {\left\{ \begin{array}{ll} \mathbb {P}\left[ L_t=\ell , V_t\ge \pi _t(s_t,\ell ), D_t\ge s_t \right] &{} \text{ if } \ell \ge 1\\ 1-\sum _{k\ge 0}\mathbb {P}[s_{t+1}=s_t+k] &{} \text { if }\ell = 0 \end{array}\right. } \end{aligned}$$

(Transitions to state “$-1$” should be read as transitions to state “$\,0\!$”.)  Note that a job of length $\ell$ may choose to purchase an interval of length greater than $\ell$, which would render these transition probabilities incorrect. However, this may only happen if the larger interval is more affordable. It is therefore in the scheduler’s interest to guarantee that $\pi _t(s,\cdot )$ in monotone non-decreasing in $\ell$, which incentivizes truthfulness, since this increases the amount of server-time available, without affecting revenue. Thus we restrict ourselves to this case.

It remains to define the transition rewards that correspond to the revenue earned. Formally, a transition from state $s_t$ to $s_t+\ell -1$ incurs a reward of $\pi _t(s,\ell )$, whereas a transition from state $s_t$ to $s_{t}-1$ incurs no reward. We wish to compute a policy $\pi$ in such a way as to maximize the expected cumulative revenue, given as the (possibly discounted) sum of all transition rewards in expectation.

#### Example 1

Consider the following situation, where the server is in state $s_t = 1$ and the job distribution is as follows:

$$\begin{aligned} (L_t,V_t, D_t) = {\left\{ \begin{array}{ll} (1,1,3) &{}\text {with probability } 0.3\\ (2,4,3) &{}\text {with probability } 0.5\\ (2,2,4) &{}\text {with probability } 0.2 \end{array}\right. } \end{aligned}$$

The following price menu is then posted: length 2 jobs cost 3,  while unitary length jobs have cost 1. If the job arriving is (1, 1, 3), then the state of the server at time $t+1$ is still 1 (one job completed, one job of length 1 scheduled) and the revenue extracted corresponds to the first entry of the price menu, i.e., 1. In the second case, if the job has parameters (2, 4, 3), then the server transitions to state 2, since the length 2 job is accepted at a price of 3. In the last case, the price requested by the server is too large, the job does not get allocated and the system goes to state 0 without earning any money.

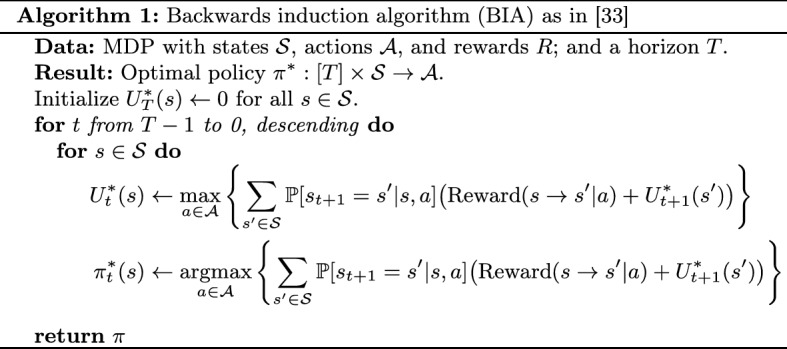



### Solving for the optimal policy with distributional knowledge

In this section, we present a modified MDP whose optimal policies can be efficiently computed, and show that these policies are optimal for the original MDP. For now, we assume that the mechanism designer is given access to the underlying distribution *Q* while, in the following sections, we show how it is possible to learn from sample enough information on *Q* to design a good strategy.

Since the problem has been modelled as a Markov Decision Process (MDP), we may rely on the wealth of literature available on MDP solutions, in particular, we can leverage the *backwards induction* algorithm (BIA) defined in Section 4.5 of the book by Putterman [33], included as Algorithm 1. However, we still need to ensure that this standard algorithm (1) runs efficiently, and (2) returns a monotone pricing policy.

Apart from the theoretical machinery of MDP and BIA, the key feature is that past prices do not contribute to future revenue insofar as the current state remains unchanged. Thus, to compute optimal current prices, we need only know the current state and expected future revenue. The idea is then to compute the optimal time-dependent policy and the incurred expected reward, for shorter horizons, then use this to recursively compute the optimal policies for longer horizons.

The total runtime of the BIA is $O(T|{\mathcal {S}}||{\mathcal {A}}|)$, where ${\mathcal {S}}$ and ${\mathcal {A}}$ denote the action and state spaces, respectively. Note that the dependence on *T* is unavoidable, since any optimal policy must be time-dependent. Recall that $\mathbb L$ and $\mathbb D$ denote the maximum values that *L* and *D* can take, respectively, and $\mathcal V$ is the set of possible values that *V* can take. Denote $\mathbb K:=\max \{\mathbb D+\mathbb L,|\mathcal V|\}$. If we define the action space naïvely, we have $|{\mathcal {S}}|=\mathbb D+\mathbb L\le \mathbb K$, and $|{\mathcal {A}}|\le \mathbb K^\mathbb L$. Thus, a naïve definition of the MDP bounds the runtime at $\mathbb K^{O(\mathbb K)}$, which is far from efficient. Requiring monotonocity only affects lower-order terms.

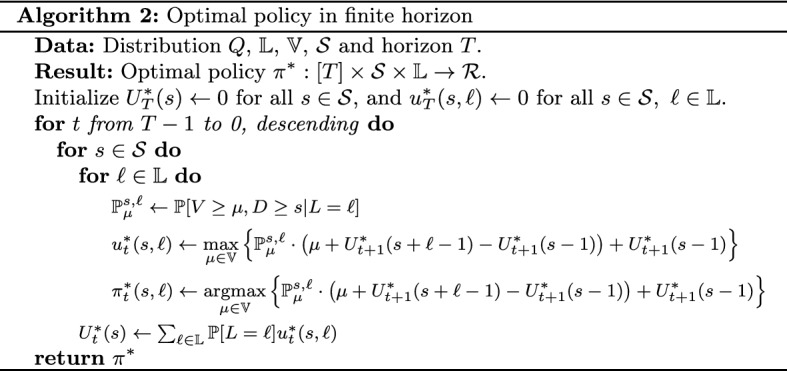



*Modified MDP* To avoid this exponential dependence, we can be a little more clever about the definition of the state space: instead of states being the possible server states, we define our state space as possible (state, length) pairs. Thus, when the MDP is in state $(s,\ell )$, the server is in state *s*, and a job of length $\ell$ has been sampled from the distribution. Our action space then is simply the possible values of $\pi _t(s,\ell )$, and the transition probabilities and rewards become:

4 $$\begin{aligned} \mathbb {P}[(s,\ell )&\rightarrow (s',\ell ')|\pi ]={\left\{ \begin{array}{ll} \mathbb {P}[V\ge \pi _t(s,\ell ),D\ge s|L=\ell ]\mathbb {P}[L'=\ell ']&{}\text {if }s'=s+\ell -1\\ \mathbb {P}[V<\pi _t(s,\ell ) \text { or } D<s|L=\ell ]\mathbb {P}[L'=\ell ']&{}\text {if }s'=s-1\\ 0 &{}\text {otherwise} \end{array}\right. } \end{aligned}$$

5 $$\begin{aligned} R((s,\ell )&\rightarrow (s',\ell ')|\pi )={\left\{ \begin{array}{ll} \pi _t(s,\ell )&{}\text { if }s'=s+\ell -1\\ 0 &{}\text {otherwise} \end{array}\right. } \end{aligned}$$

So, we get $|{\mathcal {S}}|=(\mathbb D+\mathbb L)\cdot \mathbb L\le \mathbb K^2$, $|{\mathcal {A}}|\le \mathbb K$ and therefore the runtime of the algorithm becomes $O(T\mathbb K^3)$. A full description of the procedure is given in Algorithm 2.

It remains to prove that it is correct. We begin by claiming that these two MDPs are equivalent in the following sense:

#### Lemma 1

For any fixed pricing policy $\pi :[T]\times \mathcal S\times \mathcal L\rightarrow \mathbb R$,

$$\begin{aligned} {U^\pi _{t}(s)}={{\,\mathrm{\mathbb E}\,}} _{L}\left[ {u^\pi _{t}(s,L)}\right] , \forall t \in T , \ s \in \mathcal S, \end{aligned}$$

where the ${U^\pi _{t}(\cdot )}$’s are as in (2), and the ${u^\pi _{t}(\cdot ,\cdot )}$’s are from the modified MDP.

#### Proof

We prove this result by induction on *t*; the statement is trivially true for $$t=T$$ since in that case everything is zero, so we focus on what happens for a generic $$t<T, $$ knowing that $${{\,\mathrm{\mathbb E}\,}} _{L'}\left[ {u^\pi _{t+1}(s,L')}\right] ={U^\pi _{t+1}(s)}$$ for all *s*. For the fixed policy $$\pi $$, we define $$\varvec{p}^\ell _{t,s}:=\mathbb {P}[V\ge \pi _t(s,\ell ), D\ge s|L=\ell ]$$. Then,

$$\begin{aligned} {{\,\mathrm{\mathbb E}\,}}_{L}\left[ {u^\pi _{t}(s,L)}\right]&=\sum _{\ell \in \mathcal L}\mathbb {P}[L=\ell ]{u^\pi _{t}(s,\ell )}\\&=\sum _{\ell \in \mathcal L}\mathbb {P}[L=\ell ]\Big ( \pi _t(s,\ell )\varvec{p}^\ell _{t,s} + \varvec{p}^\ell _{t,s} {{\,\mathrm{\mathbb E}\,}} _{L'}\left[ {u^\pi _{t+1}(s+\ell -1,L')}\right] \\&\qquad \qquad \qquad \qquad \qquad \ \ \ + (1-\varvec{p}^\ell _{t,s}){{\,\mathrm{\mathbb E}\,}}_{L'}\left[ {u^\pi _{t+1}(s-1,L')}\right] \Big )\\&=\sum _{\ell \in \mathcal L}\mathbb {P}[L=\ell ]\Big (\pi _t(s,\ell )\varvec{p}^\ell _{t,s} + \varvec{p}^\ell _{t,s} {U^\pi _{t+1}(s+\ell -1)}\\&\qquad \qquad \qquad \qquad \qquad \qquad \qquad + (1-\varvec{p}^\ell _{t,s}){{U^\pi _{t+1}(s-1)}}\Big )\\&= {{\,\mathrm{\mathbb E}\,}}_{\mathcal X}\left[ \textsf {Rev}_t(\pi ,\mathcal X) + {U^\pi _{t+1}(S_{t+1}(S_t,\mathcal X))}\,|\,S_t=s\right] \\&=: {U^\pi _{t}(s)}. \end{aligned}$$

Note that in the second equality we just expanded the ${u^\pi _{t}(s,\ell )}$ term conditioning on the transition at time *t*, while the following equality follows from the inductive hypothesis. The last two inequalities then follow from the definitions in Eqs. (1) and (2). $\square$

This lemma, however, does not suffice on its own, as agents may behave strategically by overreporting their length, if the prices are not increasing. This would alter the transition probabilities, breaking the analysis. In Sect. 3.5 it is proved that, under some natural assumption on the probability distribution, this can not happen: the optimal policy for non-strategic agents is monotone, and therefore truthful.

### Infinite time horizon

Algorithm 2 does not allow us to immediately compute a solution for the infinite discounted horizon case. However, we can exploit the discounting factor on the revenues to obtain an approximation of the infinite optimum: it suffices to consider the truncated problem up to a certain sufficiently large *T* and solve it optimally using the algorithm presented above. Formally, we have the following Lemma.

#### Lemma 2

For any $\varepsilon >0$ and $T\ge \log _\gamma \left( \varepsilon (1-\gamma )/\mathbb V\right)$, let $\pi$ be the pricing policy computed by the finite-horizon algorithm up to time *T*. Let $\bar{\pi }$ be the time-independent pricing policy such that $\bar{\pi }(\cdot ,\cdot ):=\pi _0(\cdot , \cdot )$. Then the expected performance of the optimal policy in the infinite horizon is within an additive $\varepsilon$ of expected performance of $\bar{\pi }$.

#### Proof

Note that in order to compute policy $\pi$ it is straightforward to add the discount factor to Algorithm 2. Let $\pi ^*$ be the Bayes-optimal infinite-horizon strategy — which is known to be time-independent — and let $\pi$ be as in the statement (where we set $\pi _t(s,\ell )=\infty$ for all $t>T$). Then, in expectation over time 0 through *T*, pricing as $\pi$ yields greater revenue than following $\pi ^*$. Conversely, in expectation over all time, pricing as $\pi ^*$ yields greater revenue than $\pi$. However, after time *T*, the maximum possible revenue due to any policy is

$$\begin{aligned} \textstyle \sum _{t=T}^\infty \gamma ^t\cdot \mathbb V= \gamma ^T \cdot \mathbb V\cdot \left( \tfrac{1}{1-\gamma }\right) \ \le \ \varepsilon \end{aligned}$$

And so the difference in revenue due to following $\pi$ or $\pi ^*$ is at most $\varepsilon$, since *T* is sufficiently large.

It remains to show that $\bar{\pi }$ performs better than $\pi$ overall. Let $\pi ^i$ be the policy which agrees with $\pi _0$ for all $t\le i$, then equals $\pi _{t-i}$ for $t>i$. Observe that, $\pi ^1$ is optimal in expectation over the interval $[1,T+1]$, and is equivalent to $\pi =\pi ^0$ for the first step. Therefore, $\pi ^1$ performs better than $\pi$. Similarly, we can argue $\pi ^{i+1}$ performs better than $\pi ^{i}$ over the interval $[i,T+i]$ and equally before, hence performs better overall.

Thus, we have a sequence of policies $\pi =\pi ^0,\,\pi ^1,\,\pi ^2,\,\dotsc$ converging to $\bar{\pi }$, and whose expected revenue is monotone nondecreasing along the sequence. Therefore, the expected revenue due to $\bar{\pi }$ is greater than that of $\pi$, which is an $\varepsilon$ additive-approximation to the optimal policy. $\square$

Therefore, we have reduced the infinite discounted horizon problem to the finite one. The discount factor $\gamma$ can be easily inserted in all proofs of the paper where needed without affecting the results. We remark that this truncation procedure is analogous to the classical value iteration technique [33].

### Approximation algorithm for continuously supported values

Similarly to what we have done in the previous section, we analyse how to generalize Algorithm 2. Note that it assumes that the *value* of the jobs (*V*) is discretely supported, and the running time depends on $|\mathcal V|$. In this section, we analyze the error incurred by discretizing the space of possible values and then computing the optimal policy.

Let $\eta >0$ be some desired small grid size, and suppose we only allow ourselves to set prices which are multiples of $\eta$. We claim that this incurs a small loss to the total revenue. It implies that the results for the finite and infinite discounted problem can be applied also in this case, paying a small additive error term.

Recall that $\varvec{p}^\ell _{s}({\mu }):=\mathbb {P}[V\ge {\mu },D\ge s|L=\ell ]$, ${U^*_{t}(s)}={{\,\mathrm{\mathbb E}\,}}_{L}\left[ {u^*_{t}(s,L)}\right]$, and

$$\begin{aligned} {u^*_{t}(s,\ell )}:= \max _{{\mu }\in \mathbb R}\left[ \varvec{p}^\ell _s({\mu })\big ({\mu }+{U^*_{t+1}(s+\ell -1)}\big ) + (1-\varvec{p}^\ell _s({\mu })){U^*_{t+1}(s-1)}\right] \end{aligned}$$

Similarly, we can define ${U^*_{t,\eta }(s)}$ and ${u^*_{t,\eta }(s,\ell )}$, restricting the maximum to choosing ${\mu }$ from multiples of $\eta$. In the following we use $\eta \cdot \mathbb {Z}$ to refer at the grid of integer multiples of $\eta .$

#### Lemma 3

The following inequality holds for all *s* and *t*:

$$\begin{aligned} |{U^*_{t}(s)}-{U^*_{t,\eta }(s)}|\le (T-t)\eta . \end{aligned}$$

#### Proof

We show this Lemma by induction on the value of *t*, decreasing. To simplify the notation, define for all *t*.

$$\begin{aligned} \varDelta _t = \max _s |{U^*_{t}(s)}-{U^*_{t,\eta }(s)}|. \end{aligned}$$

For $t=T+1$ the claimed inequality follows trivially, since $\varDelta _{T+1}=0$. We are left with bounding inductively the value of $\varDelta _t$. By the usual trick of conditioning with respect to the outcome of the transition at time *t* and some algebraic manipulation, we get that

6 $$\begin{aligned}&{u^*_{t,\eta }(s,\ell )}\nonumber \\&= \max _{{\mu }\in \eta \cdot \mathbb Z}\left[ \varvec{p}^\ell _s({\mu })\big ({\mu }+{U^*_{t+1,\eta }(s+\ell -1)}\big ) + (1-\varvec{p}^\ell _s({\mu })){U^*_{t+1,\eta }(s-1)}\right] \nonumber \\&\ge \max _{{\mu }\in \eta \cdot \mathbb Z}\Big [\varvec{p}^\ell _s({\mu })\big ({\mu }+{U^*_{t+1}(s+\ell -1)}-\varDelta _{t+1}\big ) +\nonumber \\& \quad+(1-\varvec{p}^\ell _s({\mu }))({U^*_{t+1}(s-1)}-\varDelta _{t+1})\Big ] \nonumber \\&=-\varDelta _{t+1}+\max _{{\mu }\in \eta \cdot \mathbb Z}\left[ \varvec{p}^\ell _s({\mu })\big ({\mu }+{U^*_{t+1}(s+\ell -1)}-{U^*_{t+1}(s-1)}\big ) + {U^*_{t+1}(s-1)}\right] \end{aligned}$$

Now, let ${\mu }^*$ be the optimizer of this right hand side over $\mathbb R$ (where the value would attain ${u^*_{t}(s,\ell )}$), and $\hat{\mu }$ be ${\mu }^*$ rounded *down* to the nearest multiple of $\eta$. Then, since $\varvec{p}^\ell _s(\cdot )$ is nonincreasing,

7 $$\begin{aligned} \varvec{p}^\ell _s(&\hat{\mu })\big (\hat{\mu }+{U^*_{t+1}(s+\ell -1)}-{U^*_{t+1}(s-1)}\big ) + {U^*_{t+1}(s-1)}\nonumber \\&\ge \varvec{p}^\ell _s({\mu }^*)\big ({\mu }^*-\eta +{U^*_{t+1}(s+\ell -1)}-{U^*_{t+1}(s-1)}\big ) + {U^*_{t+1}(s-1)}\nonumber \\&= {u^*_{t}(s,\ell )}- \eta \cdot \varvec{p}^\ell _s({\mu }^*) \end{aligned}$$

Thus combining equations (6) and (7), we get

$$\begin{aligned} {u^*_{t,\eta }(s,\ell )}\ \le \ {u^*_{t}(s,\ell )}\ \le \ {u^*_{t,\eta }(s,\ell )}+ \eta + \varDelta _{t+1} \end{aligned}$$

From which we conclude, by taking the expectation over $\ell$ and using the inductive assumption, that $\varDelta _t\le (T-t)\eta$, as desired. $\square$

#### Corollary 1

Let ${U^*_{}(\cdot )}$ and ${U^*_{\infty ,\eta }(\cdot )}$ be defined as above, but for the infinite horizon discounted, then $|{U^*_{}(s)}-{U^*_{\infty ,\eta }(s)}|\le \eta /(1-\gamma )\ \forall s$.

#### Proof

As shown in the previous section, it suffices to perform the analysis in the finite horizon, while taking the discount factor into account, then take the limit as $T\rightarrow \infty$. The same calculations as above gives

$$\begin{aligned}&{u^*_{t,\eta }(s,\ell )}\\ {}&\ge -\varDelta _{t+1} + \max _{{\mu }\in \eta \mathbb Z}\left[ \varvec{p}^\ell _s({\mu })\left( {\mu }+ \gamma {U^*_{t+1}(s+\ell -1)} - \gamma {U^*_{t+1}(s-1)} \right) + \gamma {U^*_{t+1}(s-1)} \right] \\ {}&\ge {u^*_{t+1}(s,\ell )}- \eta - \gamma \varDelta _{t+1} \end{aligned}$$

Summing the $\varDelta$’s and taking $T\rightarrow \infty$, we get ${u^*_{\infty ,\eta }(s,\ell )}\ge {u^*_{}(s,\ell )}- \eta /(1-\gamma )$ as desired. $\square$

### Monotonicity of the optimal pricing policies

Recall that the solution of the more efficient MDP formulation is only correct if we show that it is always monotone without considering the strategic behaviour of agents, ensuring incentive compatibility of the optimum.

An optimal monotone strategy cannot be obtained for all distributions on *L*, *V*,  and *D*. As an example, for any distribution where a job’s value is a deterministic function of their length, the optimal policy is to price discriminate by length. If this function is not monotone, the optimum won’t be either. To this end, we introduce the following assumption, which we will discuss below, and which will imply monotonicity of the pricing policy.

#### Assumption 1

*The quantity* $\tfrac{\mathbb {P}[V\ge {\mu }',D\ge s|L=\ell ]}{\mathbb {P}[V\ge {\mu },D\ge s|L=\ell ]}$ is monotone non-decreasing as $\ell$ grows, for any state *s* and $0\le {\mu }<{\mu }'$ fixed.

This is not a natural, or immediately intuitive assumption. However, we will show that it is satisfied if the valuation of jobs follows a log-concave distribution which is parametrized by the job’s length, and where the valuation is (informally) positively correlated with this length. Log-concave distributions are also commonly referred to as distributions possessing a *monotone hazard rate*, and it is common practice in economic settings to require this property of the agent valuations.

#### Lemma 4

Let, $V_\ell ^s$ denote the marginal r.v. *V* conditioned on $L=\ell$ and $D\ge s$. Let *Z* be a continuously-supported random variable, and $\rho _1^s\le \rho _2^s\le \cdots \in \mathbb R$. If $V_\ell ^s$ is distributed like $\rho _\ell ^s \cdot Z$, $\left\lfloor \rho _\ell ^s \cdot Z\right\rfloor$, $Z+\rho _\ell ^s$, or $\left\lfloor Z+\rho _\ell ^s\right\rfloor$, then Assumption 1 is satisfied if *Z* is log-concave, or if the $\rho$’s are independent of $\ell$.

A discussion of log-concave random variables and a proof of this fact is given in “Appendix”. Many standard (discrete) distributions are (discrete) log-concave random variables, including uniform, Gaussian, logistic, exponential, Poisson, binomial, etc. These can be proved to be log-concave from the discussion in “Appendix”. In the above, the $\rho$ terms represent a notion of spread or shifting, parametrized by the length, indicating some amount of positive correlation.

It remains to show the price monotonicity under the above assumption. First, we begin with the following, which holds for arbitrary distributions.

#### Lemma 5

Let ${U^*_{t}(s)}$ be the expected future revenue earned starting at time *t* in state *s*, for the optimal policy computed by Algorithm 2. Then the function $s\mapsto {U^*_{t}(s)}$ is monotone non-increasing in *s* for any *t* fixed.

#### Proof

The proof is by induction on decreasing time. At time $t=T$, there is no future revenue and ${U^*_{T}(s)}=0$, so the inductive claim follows trivially. Suppose now that the inductive claim holds at time $t+1$. It suffices to show that this holds for each ${u^*_{t}(s,\ell )}$, since ${U^*_{t}(s)}$ is simply their expectation. Let $\pi ^*_{t}$ be the optimal pricing policy computed for the time *t* by the Algorithm 2. Since the function ${\mu }\mapsto \mathbb {P}[V\ge {\mu }\ \text {and}\ {\mathcal {E}}]$, for any event ${\mathcal {E}}$, is left-continuous in the variable ${\mu }$, we may define, for every $\ell \in \mathcal L$ and $s \in \mathcal S,$

$$\begin{aligned} {\mu }_s':= \max \big \{ {\mu }: \mathbb {P}[V\ge {\mu },D\ge s|L=\ell ]\,\ge \,\mathbb {P}[V\ge \pi ^*_t(s+1,\ell ), D\ge s+1|L=\ell ] \big \} \end{aligned}$$

We must have ${\mu }'\ge \pi ^*_t(s+1,\ell )$, as $\mu =\pi ^*_t(s+1,\ell )$ is in the set. Now, letting $\varvec{p}:= \mathbb {P}[V\ge \pi ^*_t(s+1,\ell ), D\ge s+1|L=\ell ]$, we have

$$\begin{aligned}{u^*_{t}(s+1,\ell )}\\=& \varvec{p}\cdot \pi ^*_t(s+1,\ell ) + \varvec{p}\cdot {{U^*_{t+1}(s+\ell )}}+ (1-\varvec{p}){{U^*_{t+1}(s)}}\\&\quad\le \varvec{p}\cdot \pi ^*_t(s+1,\ell ) + \varvec{p}\cdot {{U^*_{t+1}(s+\ell -1)}}+ (1-\varvec{p}){{U^*_{t+1}(s-1)}}&{\small {\text {(by induction)}}}\\&\quad \le \varvec{p}\cdot \Big ( \pi ^*_t(s+1,\ell ) + {U^*_{t+1}(s+\ell -1)} - {U^*_{t+1}(s-1)}\Big )_+ + {U^*_{t+1}(s-1)}\\&\quad \le \mathbb {P}[V\ge {\mu }_s', D\ge s]\cdot \Big ({\mu }_s' + {U^*_{t+1}(s+\ell -1)} - {U^*_{t+1}(s-1)} \Big )_+ + {U^*_{t+1}(s-1)}\\&\quad\le {u^*_{t}(s,\ell )}&{\small {\text {(subopt. price)},}} \end{aligned}$$

where $(x)_+:= \max \{x,0\}$. The first inequality holds by the induction hypothesis, the second is by definition of $(\cdot )_+$, the third by the definition of ${\mu }_s'$, and in the last, from the fact that ${\mu }_s'$ is a (possibly) suboptimal pricing policy for the state *s* at time *t*. Note that this last inequality requires that the 0 value be feasible in the max, which it is, by setting $\mu '$ arbitrarily large. $\square$

This lemma ensures that overselling time on the server can only hurt the mechanism. This allows us to conclude.

#### Lemma 6

If the distribution on job parameters satisfies Assumption 1, then for all $\ell ,s,t$, we have $\pi ^*_t(s,\ell )\le \pi ^*_t(s,\ell +1)$.

#### Proof

Let $\varvec{p}^\ell _s({\mu }):= \mathbb {P}[V\ge {\mu }, D\ge s|L=\ell ]$, fix *s*, *t*, and $\ell$, and let ${\mu }_0$ be equal to the optimal price $\pi ^*_t(s,\ell )$. Observe that ${\mu }_0$ maximizes the expression

$$\begin{aligned} \varvec{p}^{\ell }_s({\mu })\big ( {\mu }+ {U^*_{t+1}(s+\ell -1)} - {U^*_{t+1}(s-1)} \big ) + {U^*_{t+1}(s-1)} \end{aligned}$$

For simplicity, let $\varDelta _\ell := {U^*_{t+1}(s+\ell -1)} - {U^*_{t+1}(s-1)}$, and so for any ${\mu }\ne {\mu }_0$,

$$\begin{aligned} 0&\le \varvec{p}^{\ell }_s({\mu }_0)\big ( {\mu }_0 + \varDelta _\ell \big ) - \varvec{p}^{\ell }_s({\mu })\big ( {\mu }+ \varDelta _\ell \big )\\&= \Big (\varvec{p}^{\ell }_s({\mu }_0) - \varvec{p}^{\ell }_s({\mu })\Big )\big ( {\mu }_0 + \varDelta _\ell \big ) + \varvec{p}^{\ell }_s({\mu })\big ({\mu }_0 - {\mu }\big ) \end{aligned}$$

Note that, as discussed in the proof of the previous lemma, $\mu _0+ \varDelta _\ell \ge 0$, as otherwise it would be beneficial to set $\pi _t^*(s,\ell )\leftarrow \infty$. The above inequality is then equivalent to

$$\begin{aligned} \frac{\varvec{p}^{\ell }_s({\mu }) - \varvec{p}^{\ell }_s({\mu }_0)}{\varvec{p}^{\ell }_s({\mu })} \le \frac{{\mu }_0-{\mu }}{{\mu }_0 + \varDelta _\ell } \quad \iff \quad \frac{\varvec{p}^{\ell }_s({\mu }_0)}{\varvec{p}^{\ell }_s({\mu })} \ge 1- \frac{{\mu }_0-{\mu }}{{\mu }_0 + \varDelta _\ell } \end{aligned}$$

We wish to show that, if ${\mu }\le {\mu }_0$, then as $\ell$ increases, the above inequality still holds. This would imply that the price ${\mu }_0=:\pi _9^*(s,\ell )$ gives better return than ${\mu }$ for jobs of length $\ell +1$, implying that the optimal price must be at least $\pi _t^*(s,\ell )$, which is our desired goal.

Now, by assumption 1, the left-hand side is non-decreasing in $\ell$, so it remains to show that the right-hand-side is non-increasing in $\ell$. The only changing term is $\varDelta _\ell$, which by Lemma 5, is non-increasing in $\ell$. Since it is in the denominator of a subtracted, non-negative term, we have our desired result. $\square$

So, putting together Lemma 6 with the work done in the previous sections, we have the following theorem.

#### Theorem 1

The online server pricing problem admits an optimal monotone pricing strategy when the variables *L*, *V*, and *D* satisfy Assumption 1. In addition,

1. In the finite horizon setting, when $\mathcal V$ is finitely supported, an exact optimum can be computed in time $O(T\mathbb K^3)$.
2. In the infinite horizon setting, when $\mathcal V$ is finitely supported, for all $\varepsilon >0$, an $\varepsilon$-additive-approximate policy can be computed in time
  $$\begin{aligned} O\left( \mathbb K^3 \log _\gamma \left( \tfrac{\varepsilon (1-\gamma )}{\mathbb V}\right) \right) \le O\left( \tfrac{\mathbb K^3}{1-\gamma }\ln \left( \tfrac{\mathbb V}{\varepsilon (1-\gamma )}\right) \right) \end{aligned}$$
3. In the finite horizon setting, when *V* is continuously supported, for all $\eta >0$, an $\eta T$-additive-approximate policy can be computed in time $O(T\mathbb K^2\mathbb V/\eta )$.

### Concentration bounds on revenue for online scheduling

In this section, we show that the revenue of arbitrary policies concentrates around their mean. In particular it holds true for the optimal or approximately optimal strategies described above. This will also allow us to argue later that, if we have an estimate $\hat{Q}$ of *Q*, then execute Algorithm 2 given the distribution $\hat{Q}$, then the output policy will perform well with respect to *Q*, both in expectation, and with high probability. To show this concentration, we consider the *Doob* or *exposure* martingale of the cumulative revenue function introduced in Sect. 2. Define

8 $$\begin{aligned} R_i^\pi :={{\,\mathrm{\mathbb E}\,}}_{}\left[ \textsf {CmlRev}_T(\pi ,{\mathcal {X}})|{\mathcal {X}}_1,\,\dotsc ,\,{\mathcal {X}}_i\right] \end{aligned}$$

where the ${\mathcal {X}}_i$’s are jobs in the sequence ${\mathcal {X}}$ and the expected value is taken with respect to $\mathcal X_{i+1}, \dots \mathcal X_T$. Thus, $R_0^\pi$ is the expected cumulative revenue, and $R_T^\pi$ is the random cumulative revenue. To formally describe this martingale sequence, we introduce and formalize some previous notations. Recall that $\mathcal X_1 ,\, \mathcal X_2,\, \dotsc$ is a sequence of jobs sampled *i.i.d.* from an underlying distribution *Q*. Fix a pricing policy ${\pi :[T]\times \mathcal S\times \mathcal L\rightarrow \mathbb R}$. Note that the state at time *t* is a random variable depending on both the (deterministic) pricing policy and the (random) $\mathcal X_1, \dots , \mathcal X_{t-1}$. We denote it $S_t(\pi ,{\mathcal {X}})$, or $S_t$ for short. Formally, suppose $\mathcal X_t=(V_t,L_t,D_t)$, then $S_{t+1}(\pi ,\mathcal X)=S_{t}(\pi ,\mathcal X)-1$ if either $V_t<\pi _t(S_t,L_t)$ or $D_t<S_t$, and otherwise $S_{t+1}(\pi ,\mathcal X)=S_t(\pi ,\mathcal X)+L_t-1$. Furthermore, let $\textsf {Rev}_t(\pi ,\mathcal X)$ be equal to 0 in the first case above (the *t*-th job is not scheduled), and $\pi _t(S_t,L_t)$ otherwise. Thus, $S_t(\pi ,\mathcal X)$ and $\textsf {Rev}_t(\pi ,\mathcal X)$ are functions of the random values $\mathcal X_1,\,\dotsc ,\,X_t$ for $\pi$ fixed. Note that $\textsf {Rev}_t$ implicitly depends on $S_t$. Let $\mathcal X_{>i}:=(\mathcal X_{i+1},\mathcal X_{i+2}, \ldots )$ and $\mathcal X_{\le i}:=(\mathcal X_1, \ldots \mathcal X_i)$. Recalling that $\textsf {CmlRev}_T(\mathcal X,\pi )=\sum _{t=1}^T\textsf {Rev}_t(\mathcal X,\pi )$, we have

9a $$\begin{aligned} R_i^\pi&= \sum _{t=0}^i \textsf {Rev}_t(\pi ,\mathcal X) + {{\,\mathrm{\mathbb E}\,}}_{\mathcal X_{>i}}\left[ \textstyle \sum _{t=i+1}^T \textsf {Rev}_t(\pi ,\mathcal X)\;\big |\;S_{i+1}(\pi ,\mathcal X_{\le i})\right] \end{aligned}$$

9b $$\begin{aligned}&= \left( \textstyle \sum _{t=0}^i \textsf {Rev}_t(\pi ,\mathcal X_{\le t})\right) + {U^\pi _{i+1}(S_{i+1}(\pi ,\mathcal X_{\le i}))} \end{aligned}$$

We wish to show that $\textsf {CmlRev}(\mathcal X,\pi )$ concentrates around its mean. Since $R_0^\pi$ is the expected revenue due to $\pi$, and $R_T^\pi$ is the (random) revenue observed, it suffices to show $|R_0^\pi -R_T^\pi |$ is small, which we will do by applying Azuma-Hoeffding inequality (as in, e.g., Theorem 13.4 of [27]) after showing the bounded-differences property.

#### Theorem 2

Let $\mathcal X$ be a finite sequence of *T* jobs sampled *i*.*i*.*d*. from *Q*, and let $\pi$ be any monotone policy. Then, with probability $1-\delta$,

$$\begin{aligned} \left| \textsf {CmlRev}_T(\mathcal X,\pi ) - {{\,\mathrm{\mathbb E}\,}}_{\mathcal X'}\left[ \textsf {CmlRev}_T(\mathcal X',\pi )\right] \right| \le \mathbb V\cdot \sqrt{2\log \left( \frac{2}{\delta }\right) T}. \end{aligned}$$

in the finite horizon, while in the infinite horizon discounted,

$$\begin{aligned} \left| \textsf {CmlRev}_\infty (\mathcal X,\pi ) - {{\,\mathrm{\mathbb E}\,}}_{\mathcal X'}\left[ \textsf {CmlRev}_\infty (\mathcal X',\pi )\right] \right| \le \mathbb V\cdot \sqrt{2\log \left( \frac{2}{\delta }\right) /(1-\gamma ^2)}. \end{aligned}$$

In particular, these results hold true for the (approximately) optimal pricing strategies of Theorem 1.

#### Proof

For the finite horizon, we apply Azuma-Hoeffding inequality to the martingale $R_t^\pi$. We begin by showing the bounded differences property. Note that we do not require truthful behaviour from the jobs, since taking strategic behaviour into account for a non-monotone policy is equivalent to modifying the distribution over the jobs and making the distribution state-dependent, by increasing the length of those jobs who would rather buy a longer interval. Thus,

$$\begin{aligned}&\left| R^\pi _{t+1}-R^\pi _{t}\right| \\ {}&\quad =\Big |\textstyle \sum _{\tau =0}^{t+1} \textsf {Rev}_\tau (\pi ,\mathcal X) + {{\,\mathrm{\mathbb E}\,}}_{\mathcal X_{>{t+1}}}\left[ \textstyle \sum _{\tau =t+2}^T \textsf {Rev}_\tau (\pi ,\mathcal X)\;\big |\;S_{t+2}(\pi ,\mathcal X_{\le t+1})\right] \\&\quad \qquad \qquad -\textstyle \sum _{\tau =0}^{t} \textsf {Rev}_\tau (\pi ,\mathcal X) - {{\,\mathrm{\mathbb E}\,}}_{\mathcal X_{>{t}}}\left[ \textstyle \sum _{\tau =t+1}^T \textsf {Rev}_\tau (\pi ,\mathcal X)\;\big |\;S_{t+1}(\pi ,\mathcal X_{\le t})\right] \Big |\\&\quad =\left| \textsf {Rev}_{t+1}(\pi ,\mathcal X) - \mathbb {E}_{\mathcal X_{t+1}}[\textsf {Rev}_{t+1}(\pi ,\mathcal X)|S_{t+1}(\pi ,\mathcal X_{\le t})]\right| \le \mathbb V\end{aligned}$$

where the equalities follow by definition and the properties of conditional expectations, while the inequality on the bound on the values. With this property, we can apply Azuma-Hoeffding inequality and get

$$\begin{aligned} \left| \textsf {CmlRev}_T(\mathcal X,\pi ) - {{\,\mathrm{\mathbb E}\,}}_{\mathcal X'}\left[ \textsf {CmlRev}_T(\mathcal X',\pi )\right] \right|&\le \sqrt{2\log \left( \frac{2}{\delta }\right) (T+1)\mathbb V^2}. \end{aligned}$$

For the infinite-horizon-discounted, we observe that Eq. (9a) and (9b) becomes

$$\begin{aligned} R_i^\pi = \sum _{t=0}^i \gamma ^t\textsf {Rev}_t(\pi ,\mathcal X) + {{\,\mathrm{\mathbb E}\,}}_{\mathcal X_{>i}}\left[ \textstyle \sum _{t=i+1}^T \gamma ^t\textsf {Rev}_t(\pi ,\mathcal X)\;\big |\;S_{i+1}(\pi ,\mathcal X_{\le i})\right] \end{aligned}$$

and thus we get that $|R_t^\pi -R_{t-1}^\pi |\le \gamma ^t\mathbb V$. Therefore, with probability $1-\delta$,

$$\begin{aligned} |R_T^\pi -R_0^\pi |&\le \sqrt{2\log (2/\delta )\textstyle \sum _{t=0}^T(\gamma ^t\mathbb V)^2}\ =\ \mathbb V\cdot \sqrt{2\log (2/\delta )\textstyle \sum _{t=0}^T(\gamma ^2)^t} \end{aligned}$$

Thus, taking the limit as $T\rightarrow \infty$, we get that with probability $1-\delta$,

$$\begin{aligned} \left| \textsf {CmlRev}_\infty (\mathcal X,\pi ) - {{\,\mathrm{\mathbb E}\,}}_{\mathcal X'}\left[ \textsf {CmlRev}_T(\mathcal X',\pi )\right] \right| \le \mathbb V\cdot \sqrt{2\log \left( \frac{2}{\delta }\right) /(1-\gamma ^2)}. \end{aligned}$$

$$\square $$

## Robustness of pricing to approximate distributional knowledge

In this section, we show that results analogous to Theorems 1 and 2 may be obtained even in the case in which we do not have full knowledge of the distribution *Q*, but only an estimate $\hat{Q}$. We then show how to obtain a valid $\hat{Q}$ from samples.

### Robustness of the pricing strategy

Let’s suppose that instead of knowing the exact distribution $Q=(D,L,V)$ of the jobs, we have only access to some estimate $\hat{Q}=( \hat{D}, \hat{L}, \hat{V})$ with the following property, for some $\varepsilon >0$: $\forall s \in S, \ell \in \mathcal L\text { and }v \in \mathcal V$ it holds that

10 $$\begin{aligned} \left| \mathbb {P}(\hat{L} = \ell ,\hat{V}\ge v, \hat{D}\ge s ) - \mathbb {P}(L=\ell , V\ge v, D\ge s)\right| < \varepsilon \end{aligned}$$

For the sake of brevity, we abuse notation and denote the condition in (10) as $|Q - \hat{Q}| < \varepsilon .$ Later, we will need to estimate the value $\mathbb {P}[L=\ell , \lnot (V\ge v, D\ge s)]$, given $\hat{Q}$, that is the probability that the job has length $\ell$, but either cannot afford price *v*, or cannot be scheduled *s* slots in the future. This is equal to $$ \mathbb {P}[L=\ell ]-\mathbb {P}[L=\ell ,V\ge v,D\ge s]\ $$.

The left-hand term is equal to $\mathbb {P}[{L=\ell ,}{V\ge 0,}{D\ge 0]}$, and so we have access to both terms. The estimation error is additive, so the deviation is at most $2\varepsilon$.

Denote $\varvec{p}^\ell _{t,s}:=\mathbb {P}[V\ge \pi ^t(s,\ell ),D\ge s|L=\ell ]$, and recall that ${U^\pi _{t}(s)}$ is defined as the expected revenue from time *t* onwards, conditioning on $S_t=s$, in formula

11 $$\begin{aligned} \sum _{\ell \in \mathcal L}\mathbb {P}[L=\ell ]\Big (\varvec{p}^\ell _{t,s}\big (\pi _t(s,\ell )+{U^\pi _{t+1}(s+\ell -1)}\big )+(1-\varvec{p}^\ell _{t,s}){U^\pi _{t+1}(s-1)}\Big ). \end{aligned}$$

Let ${\hat{U}^\pi _{t}(\cdot )}$ be the same as ${U^\pi _{t}(\cdot )}$, but where the variables are distributed as $\hat{Q}$. As before, let ${U^*_{t}(\cdot )}$ be ${U^\pi _{t}(\cdot )}$ for $\pi =\pi ^*$, the Bayes-optimal policy returned by Algorithm 2, and ${\hat{U}^*_{t}(\cdot )}$ defined similarly but with respect to $\hat{Q}$. We show now that ${\hat{U}^*_{t}(\cdot )}$ is a good estimate for ${U^*_{t}(\cdot )}$.

#### Lemma 7

Let *Q*, and $\hat{Q}$ such that $|Q - \hat{Q}| < \varepsilon$.

1. In the finite horizon, $|{U^*_{t}(s)} - {\hat{U}^*_{t}(s)}|<2\varepsilon (T-t)\cdot {\mathbb {V}} {\mathbb {L}}$ for all *t*, *s*;
2. In the infinite horizon, $|{U^*_{}(s)} - {\hat{U}^*_{}(s)}|<2\varepsilon \cdot {\mathbb {L}}{\mathbb {V}} / (1-\gamma )$ for all *s*, where $U^*$ is the optimal time independent strategy.

#### Proof

Let $\pi ^*$ be the policy computed by Algorithm 2 with access to *Q*. As in Sect. 3, we denote $\varvec{p}^\ell _{t,s}:= \mathbb {P}[V\ge \pi ^*_t(s,\ell ),D\ge s|L=\ell ]$, and ${\varvec{P}}(\ell ):=\mathbb {P}_{\mathcal X}[L=\ell ]$. In an abuse of notation, denote $\hat{\varvec{p}}^\ell _{t,s}$ and $\hat{\varvec{P}}(\ell )$ the estimated values of $\varvec{p}^\ell _{t,s}$ and ${\varvec{P}}(\ell )$, respectively. We cannot estimate $\varvec{p}^\ell _{t,s}$ directly with good error bounds, but we will only need the values $\hat{\varvec{P}}(\ell )\hat{\varvec{p}}^\ell _{t,s}$ and $\hat{\varvec{P}}(\ell )(1-\hat{\varvec{p}}^\ell _{t,s})$. Now, substituting these estimates into (11), we get:

12 $$\begin{aligned} \big |{U^*_{t}(s)}&- {\hat{U}^*_{t}(s)}\big |\nonumber \\ =&\bigg |\sum _{\ell \in \mathcal L}{\varvec{P}}(\ell )\Big (\varvec{p}^\ell _{t,s}\pi ^*_t(s,\ell ) + \varvec{p}^\ell _{t,s} {U^*_{t+1}(s+\ell -1)} + (1-\varvec{p}^\ell _{t,s}){U^*_{t+1}(s-1)}\Big ) \nonumber \\&- \sum _{\ell \in \mathcal L}\hat{\varvec{P}}(\ell )\Big (\hat{\varvec{p}}^\ell _{t,s}\pi ^*_t(s,\ell ) + \hat{\varvec{p}}^\ell _{t,s}{\hat{U}^*_{t+1}(s+\ell -1)} + (1-\hat{\varvec{p}}^\ell _{t,s}){\hat{U}^*_{t+1}(s-1)}\Big )\bigg | \end{aligned}$$

To simplify this expression, we begin by showing a simple claim: let *x*, *y*, $\hat{x}$, $\hat{y}\in \mathbb R$, and let $\lambda ,\hat{\lambda }\in [0,1]$, such that $|x-\hat{x}|<\delta$, $|y-\hat{y}|<\delta$, and $|\lambda -\hat{\lambda }|<\varepsilon$. Then, using repeatedly the triangular inequality and the properties of the absolute value, we get

13 $$\begin{aligned} \big |\big (\lambda x +&(1-\lambda ) y\big )-\big (\hat{\lambda }\hat{x} + (1-\hat{\lambda }) \hat{y}\big )\big | \nonumber \\&\le \big |\big (\lambda x + (1-\lambda ) y\big )-\big (\hat{\lambda }x + (1-\hat{\lambda }) y\big )\big |+ \nonumber \\&\qquad \qquad \big |\big (\hat{\lambda }x + (1-\hat{\lambda }) y\big )-\big (\hat{\lambda }\hat{x} + (1-\hat{\lambda }) \hat{y}\big )\big |\nonumber \\&\le |\lambda -\hat{\lambda }|\cdot |x-y| + \hat{\lambda }|x-\hat{x}| + (1-\hat{\lambda })|y-\hat{y}|\nonumber \\&\le \varepsilon |x-y|+\delta \end{aligned}$$

Replacing (repeatedly, for each $\ell$) *x* and *y* in Eq. (13) with $\big (\pi ^*_t(s,\ell )+{U^*_{t+1}(s+\ell -1)}\big )$ and ${U^*_{t+1}(s-1)}$, respectively, and replacing $\lambda$ with $\varvec{P}(\ell )\varvec{p}^\ell _{t,s}$, we have

$$\begin{aligned} \left| {U^*_{t}(s)}-{\hat{U}^*_{t}(s)}\right|&\le \sum _{\ell \in \mathcal L}\bigg ( 2{\varepsilon }\cdot \sup _{\sigma }\left| \pi _t^*(\sigma ,\ell )+{U^*_{t+1}(\sigma +\ell -1)}-{U^*_{t+1}(\sigma -1)}\right| \nonumber \\ {}&\qquad \qquad + \hat{\varvec{P}}(\ell )\cdot \sup _{\sigma '}\left| {U^*_{t+1}(\sigma ')}-{\hat{U}^*_{t+1}(\sigma ')}\right| \bigg ) \end{aligned}$$

However, the argument of the supremum in left-hand terms in the summand must be at most $\mathbb V$, since if ${U^*_{t+1}(\sigma +\ell -1)}\le {U^*_{t+1}(s-1)}$, it is best to set $\pi ^*_t(\sigma )=\infty$, which makes $\varvec{p}^\ell _{t,s}=0$, putting all the weight on ${U^*_{t+1}(s-1)}$. Furthermore, we have shown in Lemma 5 that ${U^*_{t+1}(s+\ell -1)}\le {U^*_{t+1}(s-1)}$. Thus, we get

$$\begin{aligned} \left| {U^*_{t}(s)}-{\hat{U}^*_{t}(s)}\right|&\le \textstyle \sup _{\sigma '}\left| {U^*_{t+1}(\sigma ')}-{\hat{U}^*_{t+1}(\sigma ')}\right| + \textstyle \sum _{\ell \in \mathcal L} 2{\varepsilon }\cdot \mathbb V\end{aligned}$$

Inductively applying this gives $\left| {U^*_{t}(s)}-{\hat{U}^*_{t}(s)}\right| \le 2\varepsilon (T-t)\cdot {\mathbb {V}} {\mathbb {L}}$ as desired.

Let us focus now on point 2. As in the proof of Lemma 2, if *T* is sufficiently large, we may analyze the first *T* time steps as a finite horizon problem, and the remaining revenue will be negligibly small. Now, the calculation above can be reproduced with discount terms to show

$$\begin{aligned} \left| {U^*_{t}(s)}-{\hat{U}^*_{t}(s)}\right|&\le \textstyle \sup _{\sigma '}\left| \gamma {U^*_{t+1}(\sigma ')}-\gamma {\hat{U}^*_{t+1}(\sigma ')}\right| + \textstyle \sum _{\ell \in \mathcal L} 2{\varepsilon }\cdot \mathbb V\end{aligned}$$

We can apply recursively the above formula and, letting *T* tend to infinity, we have ${|{U^*_{0}(s)}-{\hat{U}^*_{0}(s)}|\le 2\varepsilon \cdot {\mathbb {L}}{\mathbb {V}} / (1-\gamma )}$. $\square$

### Learning the underlying distribution from samples

As discussed above, we show here how to compute a $\hat{Q}$ from samples of *Q*, such that $|Q-\hat{Q}|$ is small with high probability. In particular, we present a sampling procedure which respects the rules of the pricing server mechanism. When a job arrives, we only learn its length, and only if it agrees to be scheduled. Thus, we are not given full samples of *Q*, complicating the learning procedure. Thanks to the previous section, we know that a policy which is optimal with respect to $\hat{Q}$ will be close-to-optimal with respect to *Q*.

We remark, however, that the power of the results of the previous section is not exhausted by this application: one may apply directly the robustness results to specific problems in which the $\hat{Q}$ is subject to (small) noise or an approximate distribution is already known from other sources.

Let ${\mathcal {X}}=\{(L_1,V_1,D_1),\,\dotsc ,$ $\,(L_n,V_n,D_n),\}$ be an *i.i.d.* sample of *n* jobs from the underlying distribution *Q*. Note that the expectation of an indicator is the probability of the indicated event. Fix any length $\ell$, state *s*, and value *v*, using Hoeffding bound, with probability $1-\delta$, we have that

14 $$\begin{aligned} \Big | \tfrac{1}{n}\textstyle \sum _{k=1}^n {\mathbb {I}}[L_k=\ell , V_k\ge v, D_k\ge s] -\mathbb {P}[L=\ell , V\ge v, D\ge s]\Big | \le \sqrt{\tfrac{\log \left( \frac{2}{\delta }\right) }{2n}} \end{aligned}$$

*Sampling procedure* We wish to estimate the value of $\mathbb {P}[L=\ell ,V\ge v,D\ge s]$ for all choices of $\ell$, *v*, and *s*, by simply posting price menus and observing the output. Fixing *v* and *s*, we repeatedly post prices $\pi _t(s,\ell )=v$ and declare that the earliest available time is *s*, then record (1) which job accepts to be scheduled, and (2) the length of each scheduled job. Let ${\varepsilon }>0$ and $n\ge \log (2/\delta ) / (2{\varepsilon }^2)$, then by (14), the sample-average of each value have error at most ${\varepsilon }$ with probability $1-\delta$, for any one choice of $(\ell ,v,s)$.

Repeating this process for all $\le \mathbb K^2$ choices of $v\in \mathcal V$ and $s\in \mathcal S$ gives us estimates for each. Now, if we want to have the estimate hold over all choices of $\ell ,v,s$, it suffices to take the union bound over all $\le \mathbb K^3$ values (incl. $\ell$), and scaling accordingly. If we take $n\ge 3\log (2\mathbb K/\delta )/(2{\varepsilon }^2)$ samples for each of the $\le \mathbb K^2$ choices of *v* and *s*, then simultaneously for all $\ell$, *v*, and *s*, the quantity in (14) is at most ${\varepsilon }$. Therefore, we have obtained the “$|Q-\hat{Q}|<{\varepsilon }$” condition. It should be noted that, for this sampling procedure, if a job of length $\ell$ is scheduled, we must possibly wait at most $\ell$ times units before taking the next sample to clear the buffer. This blows up the sampling *time* by a factor of $O(\mathbb L)$. From Lemma 7 and Hoeffding bound (as in Theorem 4.12 of [27]) we get the following result.

#### Lemma 8

In the finite horizon, for all $\varepsilon >0$, if $n\ge 6T\mathbb K^4\log (2\mathbb K/\delta )/\varepsilon ^2$, we have that with probability $1-\delta$, $|{U^*_{t}(s)} - {\hat{U}^*_{t}(s)}|<\varepsilon$ for all *t*, *s*. In the infinite horizon, if $n\ge 6\mathbb K^4\log (2\mathbb K/\delta )/((1-\gamma )\varepsilon ^2)$, we have that with probability $1-\delta$, $|{U^*_{}(s)} - {\hat{U}^*_{}(s)}|<\varepsilon$ for all *s*.

### Performance of the computed policy

We use here the result of the previous sections to analyze the performance of the policy output by Algorithm 2 after the learning procedure. By the estimation of revenue, the best policy in estimated-expectation is near-optimal in expectation. Since revenues from arbitrary policies concentrate, we get near-optimal revenue in hindsight.

Formally, for $\varepsilon >0$, Lemma 8 gives us that if the sample-distribution $\hat{Q}$ is computed on $n\ge 6T\mathbb K^4\log (2\mathbb K/\delta )/\varepsilon ^2$ samples, then with probability $1-\delta$ over the samples, $|{U^*_{t}(s)} - {\hat{U}^*_{t}(s)}|\le \varepsilon$. Note that ${U^*_{t=0}(s=0)}$ is exactly the expected cumulative revenue of the optimal policy. For clarity of notation, denote

15 $$\begin{aligned} \textsf {ECRev}_T(\pi |Q):= {{\,\mathrm{\mathbb E}\,}}_{\mathcal X\sim Q}\left[ \textsf {CmlRev}_T(\mathcal X,\pi )\right] \end{aligned}$$

We have shown that for sufficient samples, $|\textsf {ECRev}_T(\pi ^*|Q)-\textsf {ECRev}_T(\pi ^*|\hat{Q})|<\varepsilon$, with probability $1-\delta$. This observation allows us to conclude

#### Theorem 3

(Finite Horizon) For any precision $\varepsilon >0$, consider $n\ge 24T\mathbb K^4\log (8\mathbb K/\delta )/\varepsilon ^2$. Then in time $O(T\mathbb K^3+n\mathbb L)$, we can compute a policy $\hat{\pi }$ which is monotone in length, and therefore incentive compatible, such that for any policy $\pi$, with probability $(1-\delta )$,

$$\begin{aligned} \textsf {CmlRev}_T(\mathcal X,\hat{\pi })\ge \textsf {CmlRev}_T(\mathcal X,\pi ) - 2\mathbb V\sqrt{2\log \left( \frac{8}{\delta }\right) (T+1)} - \varepsilon \end{aligned}$$

Furthermore, if the distribution over values *V* is continuous rather than discrete, we may compute in time $O(T\mathbb K^2\mathbb V/\eta +n\mathbb L)$ a monotone policy $\hat{\pi }$ such that for any policy $\pi$, with probability $1-\delta$,

$$\begin{aligned} \textsf {CmlRev}_T(\mathcal X,\hat{\pi })\ge \textsf {CmlRev}_T(\mathcal X,\pi ) - 2\mathbb V\sqrt{2\log \left( \frac{8}{\delta }\right) (T+1)} - \varepsilon - \eta T \end{aligned}$$

#### Proof

We have chosen $n\ge 6T\mathbb K^4\log (2\mathbb K/(\delta /4))/(\varepsilon /2)^2$. Let $\pi ^*$ be the optimal policy for the true distribution *Q*. By Theorem 2, we have $|\textsf {CmlRev}_T(\mathcal X,\pi )-\textsf {ECRev}_T(\pi |Q)|<\mathbb V\sqrt{2\log (8/\delta )(T+1)}$ with probability $1-\delta /4$ for both $\pi$ and $\hat{\pi }$. Furthermore, by Lemma 8, $|\textsf {ECRev}_T(\pi |Q)-\textsf {ECRev}_T(\pi |\hat{Q})|<\varepsilon /2$ with probability $1-\delta /4$, for both $\pi =\hat{\pi }$ and $\pi ^*$. This is because from the point of view of $\hat{\pi }$, $\hat{Q}$ is the true distribution, and *Q* is the estimate. Taking the union bound over all four events above, and recalling that $\hat{\pi }$ maximizes $\textsf {ECRev}_T(\pi |\hat{Q})$, and $\pi ^*$ maximizes $\textsf {ECRev}_T(\pi |Q)$, we get the following with probability $1-\delta$:

$$\begin{aligned} \textsf {CmlRev}_T(\mathcal X,\hat{\pi })&\ge \textsf {ECRev}_T(\hat{\pi }|Q)-\mathbb V\sqrt{2\log (8/\delta )(T+1)}&\text {(concentration)}\\&\ge \textsf {ECRev}_T(\hat{\pi }|\hat{Q})-\mathbb V\sqrt{2\log (8/\delta )(T+1)}-\varepsilon /2\!\!\!\!&\text {(sample error)}\\&\ge \textsf {ECRev}_T(\pi ^*|\hat{Q})-\mathbb V\sqrt{2\log (8/\delta )(T+1)}-\varepsilon /2\!\!\!\!&\text {(optimality)}\\&\ge \textsf {ECRev}_T(\pi ^*|Q)-\mathbb V\sqrt{2\log (8/\delta )(T+1)}-\varepsilon \!\!\!\!&\text {(sample error)}\\&\ge \textsf {ECRev}_T(\pi |Q)-\mathbb V\sqrt{2\log (8/\delta )(T+1)}-\varepsilon \!\!\!\!&\text {(optimality)}\\&\ge \textsf {CmlRev}_T(\mathcal X,\pi )-2\mathbb V\sqrt{2\log (8/\delta )(T+1)}-\varepsilon \!\!\!\!&\text {(concentration)} \end{aligned}$$

as desired.

When *V* is continuously distributed, choose prices which are multiples of $\eta$ between 0 and $\mathbb V$, as is outlined in Sect. 3.4. $\square$

For what concerns the $\gamma$-discounted infinite horizon case, we have the following

#### Theorem 4

(Infinite Horizon, Discounted) For any precision $\varepsilon >0$, consider $n\ge 24\mathbb K^4\tfrac{\log (8\mathbb K/\delta )}{\varepsilon ^2(1-\gamma )}$. Then we can compute a policy $\hat{\pi }$ in time $O\left( \tfrac{\mathbb K^3}{1-\gamma }\ln \left( \tfrac{\mathbb V}{\varepsilon (1-\gamma )}\right) +n\mathbb L\right)$, which is monotone, and thus incentive compatible, such that for any policy $\pi$, with probability $(1-\delta )$,

$$\begin{aligned} \textsf {CmlRev}_\infty (\mathcal X,\hat{\pi })\ge \textsf {CmlRev}_\infty (\mathcal X,\pi ) - 2\mathbb V\sqrt{2\log \left( \frac{8}{\delta }\right) /(1-\gamma ^2)} - 2\varepsilon \end{aligned}$$

Furthermore, if the distribution over values *V* is continuous rather than discrete, we may compute in time $O\left( \tfrac{\mathbb K^2\mathbb V/\eta }{1-\gamma }\ln \left( \tfrac{\mathbb V}{\varepsilon (1-\gamma )}\right) +n\mathbb L\right)$ a monotone policy $\hat{\pi }$ such that for any $\pi$, with probability $1-\delta$,

$$\begin{aligned} \textsf {CmlRev}_\infty (\mathcal X,\hat{\pi })\ge \textsf {CmlRev}_\infty (\mathcal X,\pi ) - 2\mathbb V\sqrt{2\log \left( \frac{8}{\delta }\right) /(1-\gamma ^2)} - 2\varepsilon - \eta /(1-\gamma ) \end{aligned}$$

As above, this policy $\hat{\pi }$ is computed by learning $\hat{Q}$ from *n* samples as in Section 4.2, then running the modified Algorithm 2 for the estimated distribution as in Sect. 3.3. In case *V* is continuously distributed, we restrict ourselves to prices which are multiples of $\eta$ between 0 and $\mathbb V$. We recall that all these results need the distribution assumption from Sect. 3.5.

## Conclusions and future work

In this paper we studied the problem of pricing computing time on a single server when i.i.d. jobs arrive online with private types. The type of a job specifies its length, willingness to pay in order to get allocated and a hard deadline to get completed. We showed how the problem can be cast in the Markov Decision Process framework and how a non-trivial transformation of the state space makes the underlying problem computationally tractable while retaining incentive compatibility. Finally, we started the investigation of the learnability of the pricing problem, showing that polynomially many samples are enough to achieve a good approximation of the optimal pricing strategy.

Our model is simple but rich enough to capture many interesting features: strategic behaviour (e.g., the jobs might misreport their type), congestion (e.g., there is a carry-over effect in the server given by the queue of allocated jobs), and partial feedback (e.g., only partial information about the type is revealed to the mechanism). While the applicability of the model *per se* might appear limited, we believe that these features, as well as the techniques used, will foster future research on the subject. To conclude, we present some possible directions and their challenges.

*Multiple servers or jobs* The first natural extension to our model consists in considering multiple servers and multiple jobs arriving at each time step. The main challenge given by this problem is the high dimensionality of the state space (at least a number for each server describing its availability) and, possibly, of the action space (different price menus for each server). A possible way to overcome these difficulties might be to consider some heuristic to match supply with demand and then evaluate its approximation with respect to the optimal pricing scheme. Notice, moreover, the extra layer of complexity given by how to maintain truthfulness also in the job-server matching.

*Different congestion models* In our model we consider only greedy allocation; i.e., a job arrives and has to be immediately scheduled starting from the first available slot. This enforces the desirable property for the job to know immediately whether it gets allocated or not and when. This is important also in the application: often it is crucial to receive a quick, precise and *definitive* answer. A possible direction of research is to study different, more complex, allocation rules as, for example, in [1], where the job is not told immediately if it is accepted but only until a certain distance from the deadline. From a technical point of view, the main difficulty inherent in this approach is the increasing complexity of the MDP state space. Finally, we mention that a further generalization of our model, with interesting applications, might be to also include the waiting time suffered by the job in the pricing decision, and not only its deadline.

*Online learning* The bounds on the sample complexity contained in Sects. 4.2 and 4.3, although polynomial in the input, are impractical for real-life application. As already mentioned, this is mainly due to the specific structure of the feedback received by the mechanism, that is strictly weaker than the classical *full feedback* [7, 26]: the mechanism observes only the price (if any) chosen by the job in the proposed price menu, not the actual type of the job. An interesting direction of research is the investigation of the exploration-exploitation trade-offs of the problem in the regret minimization framework, where a natural benchmark to compare with is the best fixed price menu. Apart from the difficulty given by the feedback model, other challenges are offered by the combinatorial complexity of optimizing price menus (instead of single prices as in [7, 26]) and the carry-over effect given by the queue of allocated jobs (e.g., [3, 17]).

## Appendix: Log-concave distributions

In Sect. 3.5, we sought to show that if the value of a random job has a log-concave distribution, then the optimal policy will be monotone. We present here a discussion of log-concavity, both for continuous and discrete random variables, and give the proof of the monotonicity of the prices.

Formally, a function $f:\mathbb R\rightarrow \mathbb R$ is log-concave if for any *x* and *y*, and for any $0\le \theta \le 1$, $\lg f(\theta x + (1-\theta )y)\ge \theta \lg f(x) + (1-\theta )\lg f(y)$. Equivalently, $f(\theta x + (1-\theta )y)\ge f(x)^\theta f(y)^{1-\theta }$. For a discretely supported $f:{\mathbb {Z}}\rightarrow \mathbb R$, we replace this condition with $f(x)^2 \ge f(x-1)f(x+1)$, emulating the continuous definition with $\theta =\tfrac{1}{2}$. We further require that the support of *f* be “connected”.

### Definition 1

A continuous random variable *X* with density function *f* is said to be log-concave if *f* is log-concave. A discrete random variable *Y* with probability mass function *p* is said to be log-concave if *p* is discretely log-concave.

A well-known fact is that log-concave random variables also have log-concave cumulative density/mass functions. We present here a quick proof of this fact, for completeness.

### Claim 1

If *X* is a log-concave continuous r.v., then $\mathbb {P}[X\le x]$, and ${\mathbb {P}[X\ge x]}$ are log-concave functions of *x*. If *Y* is a log-concave discrete r.v. supported on $\mathbb {N}$, then $\mathbb {P}[Y\le y]$ and $\mathbb {P}[Y\ge y]$ are discretely log-concave functions of *y*.

### Proof

The continuous case is well-documented in the literature. See, for example [4]. For the discrete case, observe first that since the mass function is non-negative, and we have assumed contiguous support, the function must be single-peaked, *i.e.*, quasi-concave, as any local minimum would contradict the definition. Furthermore, the definition of log-concavity is equivalent to $\tfrac{p_y}{p_{y-1}}\ge \tfrac{p_{y+1}}{p_y}$. Repeatedly applying this and rearranging, we get

$$\begin{aligned} p_yp_{y+k} \ge p_{y-1}p_{y+k+1}\quad \forall y,k\in {\mathbb {Z}},\, k\ge 0\ . \end{aligned}$$

It remains to show that $P(y):=\sum _{-\infty }^y p_k$ is log-concave. We have

$$\begin{aligned} P(y)P(y)&= P(y-1)P(y)+ \sum _{-\infty }^y p_kp_y\\&\ge P(y-1)P(y) + \sum _{-\infty }^y p_{k-1}p_{y+1} = P(y-1)P(y+1) \end{aligned}$$

as desired. The same technique applies for the upper sum. $\square$

This will allow us to then conclude: (Lemma 4, p. 4) *Let* $V_\ell ^s$ denote the marginal r.v. *V* conditioned on $L=\ell$ and $D\ge s$. Let *Z* be a continuously-supported random variable, and $\rho _1^s\le \rho _2^s\le \cdots \in \mathbb R$. If $V_\ell ^s$ is distributed like $\rho _\ell ^s \cdot Z$, $\left\lfloor \rho _\ell ^s \cdot Z\right\rfloor$, $Z+\rho _\ell ^s$, or $\left\lfloor Z+\rho _\ell ^s\right\rfloor$, then Assumption 1 is satisfied if *Z* is log-concave, or if the $\rho$’s are independent of $\ell$.

### Proof

First, observe that

$$\begin{aligned} \mathbb {P}[V\ge {\mu },D\ge s|L=\ell ] = \mathbb {P}[V\ge {\mu }|D\ge s,L=\ell ]\cdot \mathbb {P}[D\ge s|L=\ell ]\ . \end{aligned}$$

and since we are taking ratios for *s* fixed, we can replace the joint cumulatives on *V* and *D* in the assumption, with the marginals on just *V*.

Now, if the $\rho$’s are independent of $\ell$, then the ratio remains unchanged as $\ell$ changes, satisfying assumption 1. Otherwise, we begin by analyzing the distributions given by $\rho _\ell ^s\cdot Z$ and $Z+\rho _\ell ^s$. Let $\bar{F}(x):= \mathbb {P}[Z\ge x]$, noting that $\mathbb {P}[V_\ell ^s\ge {\mu }] = \bar{F}({\mu }/\rho _\ell ^s)$ and $\bar{F}({\mu }- \rho _\ell ^s)$, for the two cases, respectively. Note that we wish to show $\mathbb {P}[V_\ell ^s\ge {\mu }']/\mathbb {P}[V_\ell ^s\ge {\mu }]$ is increasing, which is equivalent to $\log (\mathbb {P}[V_\ell ^s\ge {\mu }'])-\log (\mathbb {P}[V_\ell ^s\ge {\mu }])$ increasing.

For $V_\ell ^s\sim Z+\rho _\ell ^s$, observe that for $x'>x$ and $\rho '>\rho$, we have

$$\begin{aligned} \log \bar{F}(x-\rho ) - \log \bar{F}(x'-\rho ) \ge \log \bar{F}(x-\rho ') - \log \bar{F}(x'-\rho ') \end{aligned}$$

since $\log \bar{F}$ is a nonincreasing and concave function, by assumption. Also

$$\begin{aligned} \log \bar{F}(x/\rho ) - \log \bar{F}(x'/\rho )&\ge \log \bar{F}(x/\rho ') - \log \bar{F}(x/\rho ' + (x'-x)/\rho )\\&\ge \log \bar{F}(x/\rho ') - \log \bar{F}(x'/\rho ') \end{aligned}$$

where the first inequality is the same as the previous equation, as the second is by monotonicity. Thus we have done the continuous case.

For $V_\ell ^s\sim \left\lfloor Z+\rho _\ell ^s\right\rfloor$, we note that $\left\lfloor Z+\rho \right\rfloor \ge x$ if $Z+\rho \ge \left\lceil x\right\rceil$. So the probability is $\bar{F}(\left\lceil x\right\rceil - \rho )$. Similarly, for $V_\ell ^s\sim \left\lfloor \rho _\ell ^s\cdot Z\right\rfloor$, $\mathbb {P}\left\lfloor \rho Z\right\rfloor \ge x$ is $\bar{F}(\left\lceil x\right\rceil /\rho )$. Thus, if we assume that *x* and $x'$ are integers, the calculations above go through, as desired. $\square$

We present a final fact that justifies the use of $\left\lfloor Z\right\rfloor$-type random variables:

### Lemma 9

If *Y* is a discrete log-concave random variable, then there exists a continuous log-concave *Z* such that $Y\sim \left\lfloor Z\right\rfloor$.

### Proof

Let $P:\mathbb {Z}\rightarrow [0,1]$ be the right-hand cumulative mass function for *Y*. Then, it suffices to have $\mathbb {P}[Z\ge n] = P(n)$ for all integers *n*. Let $\phi :\mathbb R\rightarrow \mathbb R$ be the piecewise-linear function such that $\phi (-\infty )\rightarrow 0$, $\phi (\infty ) \rightarrow -\infty$, and $\phi (n) = \log (P(n))$ for all *n*. Since $\log (P)$ is a discretely concave and non-increasing function, $\phi$ must be concave and nonincreasing. We can then set *Z* to be the random variable whose density is given by $-\tfrac{\mathrm d}{\mathrm d x} \exp (\phi (x))$. $\square$
